# New Appliances and Things Medical

**Published:** 1898-10-15

**Authors:** 


### NEW APPLIANCES AND THINGS MEDICAL.

**[We shall be glad to receive, at our Office, 28 & 29, Southampton Street, Strand, London, W.C., from the manufacturers, specimens of all new preparations and appliances which may be brought out from time to time.]**

### CEREAL FOODS (MANHU BRAND).

(Irving, Son, and Jones, Liverpool; 88, Charing Cross Road, W.C.)

We have recently examined a considerable number of farinaceous foods, the specialities of the above firm, and distinguished by the registered trade name of Manhu. The list includes the following: Flour, flaked wheat, flaked oats, flaked barley, infants' food, buckwheat flour, gluten flour. The Manhu (old Hebrew for manna) flour is practically a whole-wheat flour of first-rate quality, and is distinguished from many of the whole-meal flours which now flood the market in being prepared from wheat deprived of *débris* and outer woody fibre or cellulose, instead of being ordinary flour loaded with the useless detritus of the refined varieties. The bread which is manufactured by the firm, and that which any cook can prepare herself from the same flour, possesses the qualifications of a first-rate brown bread. It is highly nutritious, palatable, and wholesome, and retains its moisture and sweetness remarkably well. The flaked oats, barley, and wheat for the making of porridge all seem of good value, and make an excellent breakfast dish in ten minutes. Manhu infants' food is not only a useful food for young children, but equally valuable for invalids and old persons. It is made from partially-malted Manhu flour, and with addition of certain valuable consti tuents of dried milk. For infants over eight months of age it should be particularly valuable.

### MELLIN'S FOOD AND OTHER SPECIALITIES.

(Mellin's Food, Limited, Peckham, London, S.E.)

Mellin's food is too well known to require further mention in these columns, but certain other specialities manufactured by the same firm and forwarded for our inspection deserve attention. In the first place, we would mention Mellin's food biscuits. These contain as a basis the food to which they owe their name. They are, in consequence, semi-digested, but, being solid, they give the teething child some satisfaction in the gnawing, and the adults something more than the semblance of a substantial meal. They are exceedingly palatable, and we recommend them to the notice not only of invalids. Mellin's emulsion of cod liver oil is another of the samples forwarded to us, but, equally with the Mellin's food, can be dismissed without further mention. Mellin's lacto-glycose-milk food, a complete food of very high nutritive value, requires a more detailed description. It is claimed to be prepared from Mellin's food and sterilised cow's milk, the casein of the milk being reduced by mechanical means during the process of manufacture to a more digestible condition. This highly digestible food should be of value in hot climates where milk as a food is indicated but cannot be consumed with safety. For infants who cannot tolerate milk in the unaltered state this should be a valuable substitute.

### HARZBACH WATER.

(Messrs. Brown Brothers and Co., 60, John Street, Glasgow.)

Harzbach water is a table and medicinal water containing, according to the analysis of Dr. E. T. Mills, 208·13 grs. of salts per gallon. His figures show that rather more than 50 per cent. of the solid constituents consist of sodium chloride. The carbonate of sodium, lithium, magnesium, and potassium account for the greater part of the remainder. The water is well gassed under a pressure of three atmospheres. The analysis indicates that the water has valuable properties for gouty and other conditions, where drastic measures are not desirable, and where the milder treatment of Homburg and Vichy has proved successful. The water is not unpleasant when taken by itself, or with wine or spirits, and as a corrective to acidity, heartburn, and gouty lithiasis it should have a useful future.

